# Sodium–Glucose Co-Transporter 2 Inhibitors’ Use in Muscular Dystrophy-Related Cardiomyopathy: Data from a Single-Center Experience

**DOI:** 10.3390/jcm15083098

**Published:** 2026-04-18

**Authors:** Maria Vittoria Matassini, Francesca Coraducci, Nastasia Mancini, Francesca Campanella, Chiara Carabotta, Matilda Shkoza, Lucia Pettinari, Michela Coccia, Antonio Dello Russo, Marco Marini

**Affiliations:** 1 Cardiology Division-Intensive Cardiac Care Unit, Cardiovascular Department, Azienda Ospedaliero Universitaria Delle Marche, 60126 Ancona, Italy; mariavittoria.matassini@ospedaliriuniti.marche.it (M.V.M.); francesca.coraducci@ospedaliriuniti.marche.it (F.C.); matilda.shkoza@ospedaliriuniti.marche.it (M.S.); 2 Cardiology Unit, Cardiovascular Department, Giuseppe Mazzini Hospital, 64100 Teramo, Italy; nastisedici@gmail.com; 3 Cardiology Department, Cannizzaro Hospital, 95126 Catania, Italy; campanellafrancesca95@gmail.com; 4 Department of Cardiology, Fondazione Policlinico Tor Vergata, University of Rome “Tor Vergata”, 00133 Rome, Italy; carabotta.chiara@gmail.com; 5 NeMO Clinical Centre for Neuromuscula Disease, 60126 Ancona, Italy; lucia.pettinari@centrocliniconemo.it (L.P.);; 6 Cardiology and Arrhythmology Clinic, Cardiovascular Department, Azienda Ospedaliero Universitaria delle Marche, 60126 Ancona, Italy; a.dellorusso@staff.univpm.it

**Keywords:** muscular dystrophy, cardiomyopathy, heart failure, sodium–glucose co-transporter 2 inhibitors

## Abstract

**Background**: Cardiac involvement represents a major determinant of morbidity and mortality in patients with muscular dystrophies (MDs). Evidence supporting guideline-directed heart failure (HF) therapy in this population remains limited. We aimed to retrospectively assess the effectiveness and tolerability of sodium–glucose co-transporter 2 inhibitors (SGLT2i) in patients with MDs and a previous history of HFrEF, HFpEF and HFmrEF and/or echocardiographic evidence of an LVEF < 50% **Methods**: In this retrospective, single-center study, we enrolled consecutive patients with MD treated with empagliflozin or dapagliflozin between October 2021 and October 2024. Comprehensive clinical, laboratory, echocardiographic, and functional data were collected at a baseline (V1) and at follow-up (V3) visit to evaluate longitudinal changes. **Results**: Twenty-four patients (mean age 42 ± 16 years; 92% male) were included, with a median follow-up of 418 ± 104 days. SGLT2i therapy was well tolerated; one patient discontinued treatment due to a urinary tract infection. LVEF significantly improved from 41 ± 5% to 44 ± 6% (*p* = 0.005). FSS decreased from 36 to 30 (*p* < 0.001), indicating improved functional capacity. Background HF therapy was intensified over time, with increased prescription of mineralocorticoid receptor antagonists (21% vs. 52%; *p* = 0.039) and β-blockers (67% vs. 91%). The interval between MD diagnosis and cardiomyopathy onset independently predicted LVEF improvement (β = 0.17; *p* = 0.012). **Conclusions**: In patients with MDs and HF, SGLT2i therapy was safe and associated with a modest but significant improvement in LVEF, reduced fatigue, and enhanced prescription of guideline-directed HF therapy. These findings support the potential role of SGLT2i in this high-risk population and warrant confirmation in larger prospective studies.

## 1. Introduction

Muscular dystrophies (MDs) encompass a broad group of genetic disorders that affect striated muscle and result in progressive weakness from degenerative muscle pathology.

In addition to skeletal muscle, cardiac muscle can be involved with variable clinical manifestations according to the genetic basis of the MDs phenotype [1]. Thanks to improvements in respiratory support and neuromuscular symptoms management, survival has extended and quality of life has improved [2]. Conversely, cardiac complications, such as heart failure (HF) or arrhythmias, are among the most common causes of morbidity and mortality in patients with MDs.

The management of cardiac disease in patients with MDs is challenging for several reasons. First, diagnosis is often difficult due to the wide heterogeneity in clinical presentation and because cardiovascular involvement can be masked by complex skeletal muscle symptoms. Second, our understanding of MD-specific cardiovascular pathogenesis remains limited. Third, no established guidelines exist for these specific subsets of patients as scientific evidence on the safety and efficacy of conventional HF therapies is still scarce. Moreover, the complexity of muscular involvement often restricts the titration of pharmacological treatments, as the long-term effects and potential adverse outcomes of standard HF therapies are poorly known and understood.

Different data suggest that cardiac disease progression in Duchenne muscular dystrophy (DMD)- and Becker muscular dystrophy (BMD)-related cardiomyopathy, but not in myotonic dystrophy type 1 (DM1), may be modified by the early introduction of conventional HF medical therapy, such as angiotensin-converting enzyme inhibitor (ACEi), angiotensin receptor blockers (ARB) and beta blockers [3,4]. However, cardiac therapy is usually underused and often initiated late in the disease course, when left ventricular ejection fraction (LVEF) is already impaired [5].

Sodium–glucose co-transporter 2 inhibitors (SGLT2i) recently emerged as a new therapeutic strategy for HF with reduced ejection fraction (HFrEF) [6,7,8] and represent the first drug class to demonstrate improved cardiovascular outcomes in patients with HF with preserved and mildly reduced ejection fraction (HFpEF and HFmrEF) earning a class I indication in this population [9,10]. The aim of our study was to retrospectively assess the effectiveness and tolerability of SGLT2i in patients with MDs (DMD, BMD and DM1) with a previous history of HFrEF, HFpEF and HFmrEF or echocardiographic evidence of a LVEF < 50%.

## 2. Materials and Methods

We retrospectively enrolled patients referred to our dedicated cardiology outpatient clinic, in collaboration with the NeuroMuscular Omnicenter (NeMO), a highly specialized tertiary care neuromuscular center, between October 2021 and October 2024, with a diagnosis of DMD, BMD, and DM1, who presented an LVEF < 50% and/or a previous history of HF (considering HFrEF, HFpEF and HFmrEF), and received a SGLT2i (either empagliflozin or dapagliflozin) according to HF guidelines [6,11]. The choice of agent was left to the discretion of the treating physician. The visit at which SGLT2i therapy was initiated was defined as the baseline visit (V1).

The baseline evaluation included detailed collection of past medical history (especially focused on cardiovascular risk factors, previous cardiovascular events, relevant comorbidities, ongoing medications, age at diagnosis of muscular dystrophy and age at diagnosis of cardiac involvement), anthropometric measurements (height and weight), vital signs (blood pressure and heart rate), 12-lead electrocardiogram, transthoracic echocardiogram, blood tests (hemoglobin, creatinine, LDL colhesterol, NT-proBNP, and glycemia), and functional assessments (walking ability and Fatigue Severity Scale [FSS]) and, when available, spirometry.

Data on background HF pharmacological therapy were systematically collected, including ACEi, ARBs, angiotensin receptor–neprilysin inhibitors (ARNIs), mineralocorticoid receptor antagonists (MRAs), and beta blockers. Information on diuretic therapy was also recorded, specifically including loop diuretics (furosemide) and thiazide diuretics (hydrochlorothiazide).

Echocardiographic evaluation was performed exclusively by two trained cardiologists, in accordance with the local protocol, to minimize inter-operator variability. Left ventricular (LV) systolic dimensions were measured with biplane volume, and the left ventricular ejection fraction (LVEF) was assessed with Simpson’s biplane method. Diastolic dysfunction was assessed following the American Society of Echocardiography recommendation [12]. Paired follow-up data, as well as any complications related to SGLT2i use (urinary tract infections, hypotension, constipation, and pruritus), were recorded. Data on cardiac implantable electronic device (CIED) implantation were also collected.

Coronary artery disease (CAD) was systematically excluded in all patients based on clinical history, cardiovascular risk factor assessment, and non-invasive testing. When clinically indicated, coronary CT angiography was performed.

Patients were re-evaluated at a follow-up visit, named “V3” and the same variables as previously described for V1 were collected. The intermediate visit (“V2”) was excluded from the analysis because it occurred within a short interval from baseline

The study was conducted in accordance with Good Clinical Practice (GCP) guidelines and the principles of the Declaration of Helsinki. All data were anonymized, and written informed consent was obtained from all patients for inclusion in the study and publication of the findings.

### Statistical Analysis

Continuous variables are reported as the mean ± standard deviation (SD) or as median and quartiles [Q1–Q3], depending on the distribution assessed using the Shapiro–Wilk test. Categorical variables are presented as counts and percentages. Paired comparisons of continuous variables before and after treatment with SGLT2 inhibitors were conducted using the paired Student’s *t*-test or the Wilcoxon signed-rank test, as appropriate. For paired categorical variables, McNemar’s test was used, and effect size was estimated using the log odds ratio with 95% confidence intervals. Correlations between continuous variables were examined using Pearson’s or Spearman’s correlation coefficients, based on data distribution. A multivariable linear regression model was built to identify independent predictors of response to SGLT2 inhibitors. All variables with clinical significance and a *p*-value < 0.1 at univariable linear regression analysis were included in the multivariable model. A two-sided *p*-value < 0.05 was considered as statistically significant. Data were analyzed using Jamovi (version 2.6.19; https://www.jamovi.org, accessed on 1 January 2026), an open-source statistical software built on the R language.

## 3. Results

A total of 26 patients were screened; two did not initiate the prescribed therapy because of poor compliance and were therefore excluded, leaving 24 patients for the final analysis. One patient discontinued treatment owing to a urinary tract infection; all others reported no adverse effects and tolerated SGLT2 inhibitors well. The baseline (V1) and last follow-up (V3) visits data are summarized in Table 1 and Table 2. The median interval between V1 and V3 visits was 418 ± 104 days.

Of the 24 patients, 8 (33%) had DM1, 8 (33%) had BMD, and 8 (33%) had DMD. At V1, nineteen (79%) were treated with empagliflozin and five (21%) with dapagliflozin. The mean age at V1 was 42 ± 16 years; 22 patients (92%) were male. The mean time from muscular dystrophy diagnosis to cardiomyopathy onset was 16 ± 12 years.

At V1, six patients (25%) had arterial hypertension, two (8%) had diabetes, seven (29%) had dyslipidemia, six (25%) obesity, and nine (37%) a history of smoking. Chronic obstructive pulmonary disease (COPD) was present in two (8%) patients; none had documented coronary artery disease (CAD). Twelve patients (50%) were on non-invasive ventilation. The prevalence of cardiovascular risk factors, COPD and most importantly CAD was unchanged at V3.

Median systolic/diastolic blood pressure at V1 was 101 (110–115)/65 (60–70) mmHg and did not differ at V3 (mean change −5 mmHg; *p* = 0.200). Dyspnoea on exertion was reported by nine patients (38%) at V1 and by only 2 (11%) at V3, a reduction that, although large, did not reach statistical significance (*p* = 0.063). Atrial arrhythmias (atrial fibrillation/flutter) were present in three patients (13%) at both visits. Of the three patients with atrial arrhythmias, two were anticoagulated; the remaining patient had low thromboembolic risk (CHA_2_DS_2_-VASc = 1), increased fall-related bleeding risk, and isolated paroxysmal AF, and was therefore not anticoagulated. Three additional patients received cardiac resynchronization therapy (CRT) during follow-up due to bradyarrhythmic problems and not for further HF or EF worsening, but the increase was not significant (*p* = 0.125).

**Figure 1:**
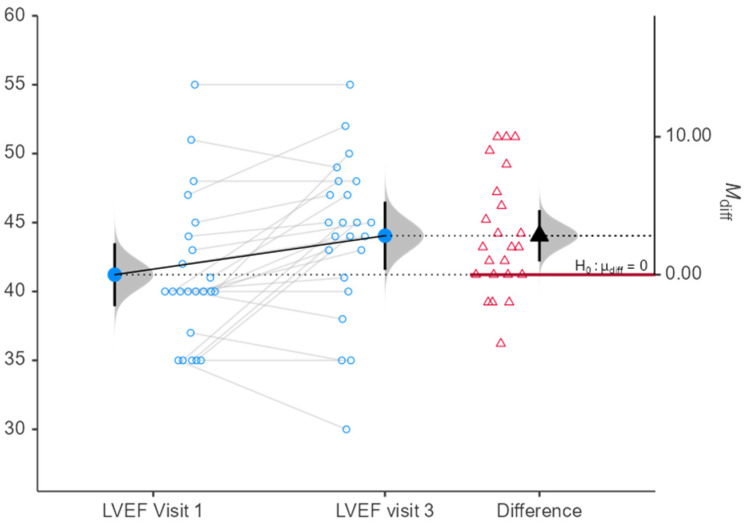
Difference in LVEF from visit 1 to visit 3.

No significant changes were observed in routine laboratory parameters. The Median NT proBNP fell by 54 pg/mL but this was not significant (*p* = 0.496). Echocardiography showed a significant improvement in LVEF, rising from 41 ± 5% at V1 to 44 ± 6% at V3 (*p* = 0.005; Figure 1).

No significant changes were observed in diastolic dysfunctions. The right ventricular function and dimensions remained stable (median TAPSE 21 mm at baseline vs. 20 mm at follow-up; *p* = 0.444). Mitral valve prolapse was noted in two patients at baseline and three at the follow-up (*p* = 0.500). The median pulmonary artery systolic pressure (PAPs) did not change (27 mmHg vs. 25 mmHg; *p* = 0.306).

Notably, a significant reduction in fatigue symptoms was noticed between visits with mean FSS decreasing from 36 at V1 to 30 at V3 (mean change −6; *p* < 0.001). Moreover, walking ability remained largely stable between V1 and V3, with only one patient experiencing a decline from full autonomy to requiring assistance of a walker.

Background heart failure therapy prescription was significantly increased from V1 to V3. The proportion of patients receiving a MRA more than doubled, rising from 5 patients (21%) at V1 to 12 patients (52%) at V3 (*p* = 0.039). Up-titration of β blockers showed a similar pattern, with 15 patients (67%) on therapy at V1 versus 21 patients (91%) at V3. In addition, five patients switched from an ACEi or ARB) to an ARNI: overall ACEi/ARB use fell from 71% at V1 to 43% at V3 (*p* = 0.031), while ARNI prevalence increased from 25% to 48% (trend *p* = 0.063).

**Figure 2:**
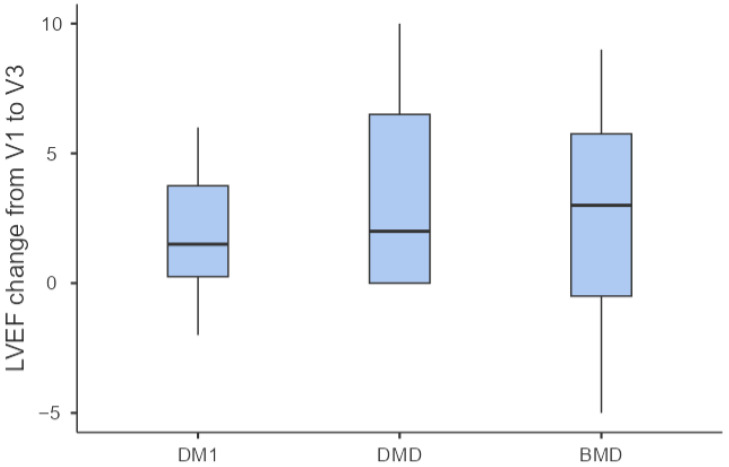
Mean improvement in LVEF from visit 1 to visit 3 across all muscular dystrophy subtypes.

The improvement in LVEF (defined as LVEF at V3—LVEF at V1) did not differ among MD subtypes (Figure 2).

In multivariable analysis (Table 3), the only independent predictor of LVEF improvement was the time interval between MD diagnosis and cardiomyopathy (CM) onset in years (beta = 0.17 and 95% CI 0.01–0.09; *p* = 0.012). Neither MD subtype nor baseline LVEF at first visit were significant predictors. The model explained 37% of the variance (R^2^ = 0.37), with no evidence of collinearity among covariates.

**Figure 3:**
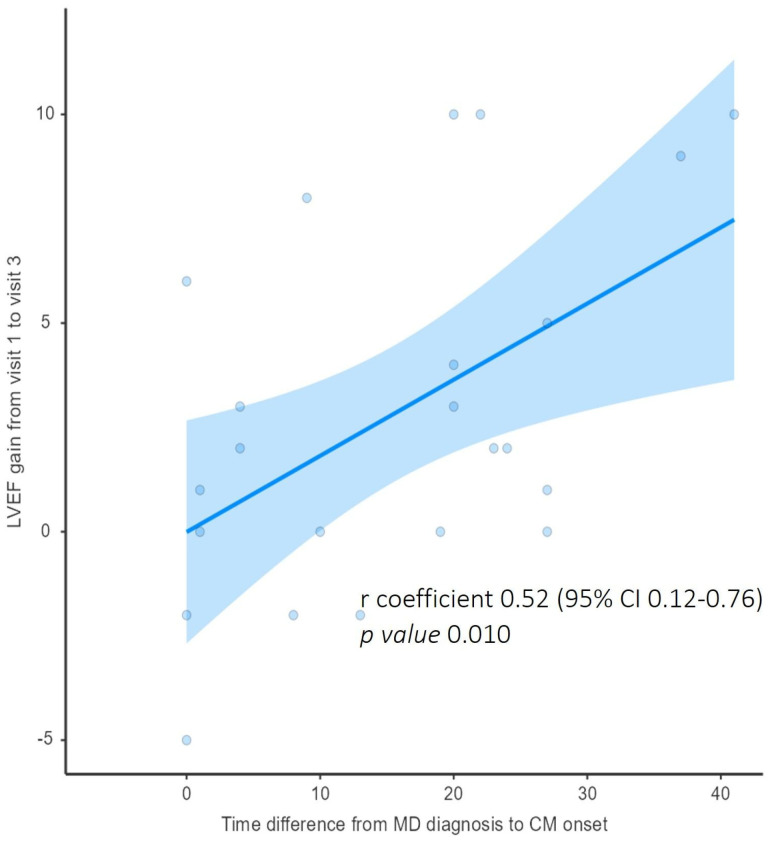
Correlation between improvement in LVEF from visit 1 to visit 3 and time interval from muscular dystrophy diagnosis and cardiomyopathy (CM) onset.

The time interval between MD diagnosis and CM onset also showed a significant, although modest, correlation with LVEF improvement between V1 and V3 (r = 0.52 and 95% CI 0.12–0.76; *p* = 0.010, Figure 3).

## 4. Discussion

Our research represents a pivotal study in the field, being one of the first studies assessing the effects of SGLT2i in MDs patients. In patients with MDs and overt HF or echocardiographic evidence of LVEF < 50%, we found that the introduction of SGLT2i was beneficial across several domains.

The most relevant finding of our study was a significant improvement in functional capacity, demonstrated by a mean reduction of six points in FSS between V1 and V3. This result is particularly remarkable given the progressive decline in muscle function typically observed in this patient population [13].

Fatigue in muscular dystrophy is inherently multifactorial and may be influenced by neuromuscular progression, respiratory status, psychological factors, and rehabilitation interventions. However, in our cohort, neuromotor status—systematically assessed by physiatrists and rehabilitation specialists at the NeMO Center—remained clinically stable throughout the follow-up. No relevant changes in neuro-rehabilitative or psychological therapies were introduced compared with the established standard of care, and ventilatory support requirements did not significantly vary. In this context of overall neuromuscular and respiratory stability, the observed improvement in FSS may reasonably be interpreted as being predominantly influenced by the cardiac component, although causality cannot be definitively established due to the study design.

The molecular pathways underlying this benefit are not fully understood; however, preclinical studies suggest that muscle weakness in HF is linked to increased protein degradation and inflammation [14], as well as impaired calcium handling [15,16]. Since SGLT2i are known to interact with sodium and glucose channels, they may contribute to enhanced calcium homeostasis and improved muscle contractility. Supporting this hypothesis, a murine model of high-salt-diet-induced HFpEF treated with SGLT2i showed a significant attenuation of HF-induced atrophy in the digitalis longus muscle [17]. In addition, recent clinical research in patients with HFrEF, whose skeletal muscle function was assessed by biopsy, showed that SGLT2i therapy was associated with anti-atrophic, pro-metabolic, and anti-inflammatory effects mediated via IL-6–kynurenine pathway [18].

Alongside the improvement in functional capacity, we found a consistent, although statistically not significant, reduction in patients reporting dyspnea on exertion.

We acknowledge that dyspnea assessment in patients with MD may be confounded by the underlying neuromuscular disorder [19]. Nevertheless, given the documented stability of neuromuscular and respiratory status during follow-up, a potential contribution of SGLT2 inhibitor therapy to the observed reduction in dyspnea cannot be excluded.

Alongside the observed improvement in fatigue, we documented a significant increase in LVEF, from a mean of 41 ± 5% at V1 to a mean of 44 ± 6% at V3 (*p* = 0.005). Although this change is modest, it is in line with previous meta-analysis on the effect of SGLT2i in patients with HF [20,21,22]. Importantly, in a relatively young population with a progressive condition such as muscular dystrophy (mean age 42 years in our cohort), even modest improvement—or, at minimum, stabilization—of systolic function represents a clinically meaningful achievement. Cardiologists should consider this favorable effect on LVEF in younger HF populations, as it may have significant implications for long-term cardiac outcomes and disease progression. In line with previous reports [21], the relatively shorter duration of cardiomyopathy in some patients may have contributed to the observed improvement in LVEF, as earlier stages of the disease are generally associated with a higher likelihood of functional recovery. However, this observation should be interpreted with caution, given the observational nature of both studies. Nonetheless, the consistency of these findings across similar studies may lend further support to our results.

Notably, the improvement in LVEF did not differ across MD subtypes. In the multivariable model, the time interval from MD diagnosis to the onset of cardiomyopathy emerged as the only independent predictor of LVEF improvement, after adjustment for baseline LVEF and MD subtype. Although the explanatory power of the model was modest (R^2^ = 0.37), this finding is supported by the positive correlation between a longer interval from MD diagnosis to cardiomyopathy onset and greater improvement in LVEF, suggesting that patients with later onset of CM experienced greater benefit from SGLT2i therapy.

To be noted, the walking ability in non-wheelchair patients at baseline did not decline significantly over time, and need for ventilatory support remained stable in all study population, thereby minimizing potential confounders in the assessment of treatment effect. Moreover, our cohort had a low prevalence of other comorbidities (such as CAD or COPD).

In our study, the initiation of SGLT2i was also associated with improved prescription of guideline-directed medical therapy, as reflected by a significant increase in the proportion of patients receiving MRA and beta-blocker therapy at V3, without a concomitant rise in diuretic use. Although not specifically investigated in our study, SGLT2i attenuates the risk of MRA-associated hyperkaliemia through an increase in the rate of sodium delivery to the Na+/K+ exchanger in distal tubules and also through the improvement of kidney function or prevention of kidney disease progression, as already reported in the literature. This favorable effect “enables” a broader MRA prescription, with secondary synergistic benefits from both HF pillars on outcomes [23].

Moreover, SGLT2i may have contributed to creating a more stable clinical condition allowing the introduction or up-titration of additional therapies potentially improving adherence. We also observed a reduction in ACEi/ARB therapy over time, which can be explained by the switch of five patients from ACEi/ARB to ARNI. Despite the existing evidence being limited, real-word data have already shown how SGLT2 use facilitates ARNI initiation and titration in general HF population [24] as in our dystrophic patients.

Overall, this pattern reflects a favorable adaptation to medical therapy, allowing a greater number of patients to benefit from cardioprotective treatments.

Last but not least, we observed no adverse effects related to SGLT2i therapy, except for one subject who discontinued empagliflozin at the very beginning of treatment due to a urinary tract infection and refused to resume therapy.

## 5. Limitations

Our study provides valuable insights but is subject to several important limitations. First, its retrospective design precludes full control over potential confounders, which may have influenced the observed outcomes. Second, the cohort is relatively small and heterogeneous, encompassing patients with distinct forms of muscular dystrophy (DMD, BMD, and DM1). While DMD and BMD share a common genetic basis, they differ markedly in terms of disease severity and the extent of cardiac involvement, and these differences are even more pronounced when DM1 is included. Furthermore, the observed improvement in LVEF cannot be attributed exclusively to iSGLT2 therapy, as patients also underwent concomitant optimization of guideline-directed medical therapy, including ARNI, beta blockers, ARM, and TRC, which may have contributed to cardiac function recovery. Therefore, the independent effect of iSGLT2 on LVEF improvement cannot be clearly disentangled, and these findings should be interpreted with caution. Despite these limitations, we believe our findings are highly relevant, as they provide preliminary evidence supporting the potential safe and effective use of SGLT2 inhibitors in this rare and underexplored population. Moreover, our data may serve as a foundation to stimulate further research and guide the design of larger, prospective studies in a field where robust evidence is urgently needed.

## 6. Conclusions

In conclusion, we found that the introduction of SGLT2i in our patients with MDs was overall well tolerated and led to a significant improvement in functional capacity, as assessed by the FSS scale, alongside with a mean 3% increase in LVEF, and it was associated with a higher prescription of background HF therapies. The time interval from MD diagnosis to CM onset was a predictor of LVEF improvement when adjusted for baseline LVEF and MD subtype, with greater LVEF gains after SGLT2i initiation observed in patients whose CM developed later following MD diagnosis. Although this study is based on a small cohort, it suggests a promising and effective use of SGLT2I in MDs patients while awaiting larger-scale evidence.

## Figures and Tables

**Table 1:** Characteristics of study population (*n* = 24) at visit 1.

Demographics and Medical History	Visit 1 (*n* = 24)
Muscular dystrophy (%)	DM1 8 (33) Duchenne 8 (33) Becker 8 (33)
SGLT2i (%)	Dapagliflozin 5 (21) Empagliflozin 19 (79)
Age (years)	42 ± 16
Sex (%)	Male 22 (92) Female 2 (8)
Time from dystrophy diagnosis to cardiomyopathy onset (years)	16 ± 12
Time from visit 1 to visit 3 (days)	418 ± 104

SGLT2i: sodium–glucose co-transporter 2 inhibitors.

**Table 2:** Difference between visit 3 and visit 1.

	Visit 1	Visit 3	Mean Difference (95% CI)	*p*-Value
Demographics and medical history	Total 24	Total 23		
Weight (kg)	76 ± 22	75 ± 22	0 (−1.98; 1.72)	0.885
Height (cm)	171 ± 9	171 ± 11	−1 (−3.17; 1.79)	0.535
BMI (kg/m^2^)	25 ± 6	25 ± 5	0.55 (−1.0; 1.35)	0.715
FSS	36 ± 10	30 ± 9	−6	<0.001
Hypertension (%)	6 (25)	7 (30)	1	0.317
Diabetes (%)	2 (8)	2 (10)	0	1.000
Dyslipidemia (%)	7 (29)	8 (35)	1	1.000
Obesity (%)	6 (25)	5 (22)	1	1.000
Smoking habit (%)	9 (37)	6 (26)	3	1.000
COPD (%)	2 (8)	1 (4)	−1	1.000
CKD (%)	1 (5)	1 (5)	0	na
Walking (%)				
Normal	12 (50)	11 (47)	−1	0.704
With helper	4 (17)	5 (22)	1	
Wheelchair	8 (33)	7 (26)	0	
OSAS (%)	4 (18)	4 (19)	2	na
NIV (%)	11 (52)	11 (52)	0	na
CAD (%)	0 (0)	0 (0)	0	na
Vitals and EKG				
SBP (mmHg)	110 [110–120]	100 [110–118]	−5 (−13.00; 2.50)	0.200
DBP (mmHg)	67 [60–74]	65 [60–70]	−5 (−10; 1.50)	0.125
HR (beats/min)	69 [55–79]	61 [57–71]	−9 (−18.00; 0.00)	0.138
Symptoms (%)				
Chest pain	0 (0)	0 (0)	0–5	na
Dyspnoea	9 (38)	4 (17)	0	0.063
Palpitations	1 (4)	1 (5)	−1	1.000
Syncope	2 (8)	1 (4)		1.000
AF/AFL (%)	3 (13)	3 (16)	0	1.000
Conduction disturbances (%)				
AVB I grade	6 (17)	4 (11)	−2	0.500
AVB II grade	0 (0)	0 (0)	0	na
AVB III grade	0 (0)	0 (0)	0	na
RBBB	4 (17)	1 (4)	−3	0.250
LBBB	8 (33)	3 (13)	−4	0.250
CIEDs (%)				
PMK	1 (4)	3 (13)	2	0.625
ICD	2 (8)	3 (13)	1	0.317
CRT	1 (4)	4 (17)	3	0.125
Echocardiography				
LVEF (%)	41 ± 5	44 ± 6	3 (0.97; 4.69)	0.005
LVEDD (mm)	54 ± 7	52 ± 6	−1 (−3.06; 1)	0.446
LVEDVi (ml/m^2^)	68 ± 14	65 ± 11	−3 (−8; 2.23)	0.225
Diastolic dysfunction (%)	12 (50)	12 (52)	0	1.000
E/e’	5 [5–12]	5 [4–9]	3 (−3; 9)	0.313
TAPSE (mm)	21 [18–25]	20 [18–23]	−1 (−3.50; 2.00)	0.444
S’ TDI (cm/s)	11 [10–12]	12 [10.25–13]	1 (−15.0; 2)	0.683
RVD1 (mm)	35 [33–39]	37 [35–41]	2 (−2.00; 4.00)	0.085
MR (%)				na
None	4 (17)	6 (26)	2	
Mild	17 (71)	16 (70)	−1	
Moderate	2 (13)	1 (4)	−1	
Severe	0 (0)	0 (0)	0	
Mitral prolapse (%)	2 (8)	3 (21)	1	0.500
PAPs (mmHg)	27 [24–29]	25 [20–31]	−2 (−6.00; 4.00)	0.306
Lab				
Glycemia (mg/dL)	89 [79–94]	74 [71–89]	−2.5 (−15.00; 14.00)	0.688
Creatinine (mg/dL)	0.66 [0.45–0.75]	0.95 [0.72–1.14]	0.20 (−0.01; −0.41)	0.062
LDL (mg/dL)	106 [92–121]	104 [93–116]	2.00 (−13.50; −18.00)	0.695
HB (g/L)	14 [14–15]	14 [13–15]	0.55 (−0.15; −1.05)	0.084
NTproBNP (pg/mL)	131 [96–209]	142 [70–300]	54 (−27; 170)	0.496
K (mEq/L)	4.0 [3.7–4.4)	4.2 [4.10–1.8]	0.2 (−0.32–0.27)	0.841
Medical therapy				
ACEi/ARB (%)	16 (71)	10 (43)	−5	0.031
ARNI (%)	6 (25)	11 (48)	5	0.063
Beta blockers (%)	15 (67)	21 (91)	6	0.031
MRA (%)	5 (21)	12 (52)	7	0.039
Diuretics (%)	3 (13)	1 (5)	2	0.500
Statins (%)	0 (0)	1 (5)	1	na
Ezetimibe (%)	5 (21)	7 (30)	2	0.625
Cardioaspirin (%)	1 (4)	0 (0)	−1	na
Antiarrhythmics (%)	1 (4)	2 (9)	1	1.000
DOAC (%)	2 (8)	2 (9)	0	1.000

ACE/ARB: ACE inhibitor/angiotensin receptor blocker, ARNI: angiotensin receptor–neprilysin inhibitor, AF/Afl: atrial fibrillation/atrial flutter, BMI: body mass index, CAD: coronary artery disease, CIEDs: cardiac implantable electronic devices, CKD: chronic kidney disease, COPD: Chronic obstructive pulmonary disease, DBP: diastolic blood pressure, DOAC: direct oral anticoagulant, FSS: Fatigue Severity Scale, HR: heart rate, LVEDD: left ventricular end-diastolic diameter, LVEDVi: left ventricular end-diastolic volume index, LVEF: left ventricular ejection fraction, MR: mitral regurgitation, MRA: mineralocorticoid receptor antagonist, NIV: non-invasive ventilation, OSAS: obstructive sleep apnea syndrome, PAPs: systolic pulmonary artery pressure, RVD1: right ventricular diameter, SBP: systolic blood pressure, and TAPSE: tricuspid annular plane systolic excursion.

**Table 3:** Multivariable analysis; predictors of LVEF change from visit 1 to visit 3.

Predictor	B	95% CI	*p*-Value	Collinearity Tests (VIF; Tolerance)
Muscular dystrophy				1.03; 0.97
Duchenne—DM1	−0.71	−5.00; 3.59	0.743	
Becker—DM1	−1.35	−5.45; 2.75	0.489	
Time from MD diagnosis to cardiomyopathy onset	0.17	0.05; 0.34	0.012	1.05; 0.95
LVEF at visit 1	−0.025	−0.057; 0.07	0.123	1.01; 0.99
R^2^ = 0.37; R = 0.61				

CI: confidence interval, DM1: myotonic dystrophy type 1, LVEF: left ventricular ejection fraction, and MD: muscular dystrophy.

## Data Availability

No new data were created.

## Abbreviations

ACEi	angiotensin-converting enzyme inhibitor
AF	atrial fibrillation
AFL	atrial flutter
ARB	angiotensin receptor blocker
ARNI	angiotensin receptor–neprilysin inhibitor
AVB	atrioventricular block
BMD	Becker muscular dystrophy
BMI	body mass index
CAD	coronary artery disease
CIED	cardiac implantable electronic device
CKD	chronic kidney disease
CM	cardiomyopathy
COPD	chronic obstructive pulmonary disease
CRT	cardiac resynchronization therapy
DBP	diastolic blood pressure
DM1	myotonic dystrophy type 1
DMD	Duchenne muscular dystrophy
DOAC	direct oral anticoagulant
EF	ejection fraction
FSS	Fatigue Severity Scale
GCP	Good Clinical Practice
HF	heart failure
HFmrEF	heart failure with mildly reduced ejection fraction
HFpEF	heart failure with preserved ejection fraction
HFrEF	heart failure with reduced ejection fraction
HR	heart rate
ICD	implantable cardioverter defibrillator
IL-6	interleukin-6
LDL	low-density lipoprotein
LBBB	left bundle branch block
LVEDD	left ventricular end-diastolic diameter
LVEDVi	left ventricular end-diastolic volume index
LVEF	left ventricular ejection fraction
MD	muscular dystrophy
MDs	muscular dystrophies
MRA	mineralocorticoid receptor antagonist
MR	mitral regurgitation
Na^+^/K^+^	sodium/potassium
NeMO	NeuroMuscular Omnicenter
NIV	non-invasive ventilation
NT-proBNP	N-terminal pro-B-type natriuretic peptide
OSAS	obstructive sleep apnea syndrome
PAPs	systolic pulmonary artery pressure
PMK	pacemaker
RBBB	right bundle branch block
RVD1	right ventricular diameter
SBP	systolic blood pressure
SD	standard deviation
SGLT2i	sodium–glucose co-transporter 2 inhibitors
TAPSE	tricuspid annular plane systolic excursion
TDI	tissue Doppler imaging
TnIhs	high-sensitivity troponin I

## References

[1] Feingold B., Mahle W.T., Auerbach S., Clemens P., Domenighetti A.A., Jefferies J.L., Judge D.P., Lal A.K., Markham L.W., Parks W.J. (2017). Management of Cardiac Involvement Associated With Neuromuscular Diseases: A Scientific Statement from the American Heart Association. Circulation.

[2] Jefferies J.L., Meune C., Lerebours G., Devaux J.-Y., Vaksmann G., Bécane H.-M. (2005). Genetic predictors and remodeling of dilated cardiomyopathy in muscular dystrophy. Circulation.

[3] Duboc D., Thomas S., Neyaz T., Ciafaloni E., Mann J.R., Staron-Ehlinger M., Beasley G.S., Romitti P.A., Mathews K.D. (2005). Effect of perindopril on the onset and progression of left ventricular dysfunction in Duchenne muscular dystrophy. J. Am. Coll. Cardiol..

[4] Conway K.M., Thomas S., Neyaz T., Ciafaloni E., Mann J.R., Staron-Ehlinger M., Beasley G.S., Romitti P.A., Mathews K.D. (2025). Prophylactic Use of Cardiac Medications and Survival in Duchenne Muscular Dystrophy. Muscle Nerve.

[5] Spurney C., Shimizu R., Hache L.P., Kolski H., Gordish-Dressman H., Clemens P.R., the CINRG Investigators (2014). CINRG Duchenne Natural History Study demonstrates insufficient diagnosis and treatment of cardiomyopathy in Duchenne muscular dystrophy. Muscle Nerve.

[6] McDonagh T.A., Metra M., Adamo M., Gardner R.S., Baumbach A., Böhm M., Burri H., Butler J., Čelutkienė J., Chioncel O. (2021). 2021 ESC Guidelines for the diagnosis and treatment of acute and chronic heart failure. Eur. Heart J..

[7] McMurray J.J.V., DeMets D.L., Inzucchi S.E., Køber L., Kosiborod M.N., Langkilde A.M., Martinez F.A., Bengtsson O., Ponikowski P., Sabatine M.S. (2019). A trial to evaluate the effect of the sodium-glucose co-transporter 2 inhibitor dapagliflozin on morbidity and mortality in patients with heart failure and reduced left ventricular ejection fraction (DAPA-HF). Eur. J. Heart Fail..

[8] Anker S.D., Butler J., Filippatos G., Marx N., Schnaidt S., Ofstad A., Ponikowski P., Pocock S., Zannad F., Packer M. (2021). Effect of Empagliflozin on Cardiovascular and Renal Outcomes in Patients With Heart Failure by Baseline Diabetes Status: Results From the EMPEROR-Reduced Trial. Circulation.

[9] Anker S.D., Butler J., Filippatos G., Ferreira J.P., Bocchi E., Böhm M., Brunner-La Rocca H.P., Choi D.J., Chopra V., Chuquiure-Valenzuela E. (2021). Empagliflozin in Heart Failure with a Preserved Ejection Fraction. N. Engl. J. Med..

[10] Solomon S.D., McMurray J.J., Claggett B., de Boer R.A., DeMets D., Hernandez A.F., Inzucchi S.E., Kosiborod M.N., Lam C.S., Martinez F. (2022). Dapagliflozin in Heart Failure with Mildly Reduced or Preserved Ejection Fraction. N. Engl. J. Med..

[11] McDonagh T.A., Metra M., Adamo M., Gardner R.S., Baumbach A., Böhm M., Burri H., Butler J., Čelutkienė J., Chioncel O. (2023). 2023 Focused Update of the 2021 ESC Guidelines for the Diagnosis and Treatment of Acute and Chronic Heart Failure. Eur. Heart J..

[12] Nagueh S.F., Smiseth O.A., Appleton C.P., Byrd B.F., Dokainish H., Edvardsen T., Flachskampf F.A., Gillebert T.C., Klein A.L., Lancellotti P. (2016). Recommendations for the Evaluation of Left Ventricular Diastolic Function by Echocardiography: An Update from the American Society of Echocardiography and the European Association of Cardiovascular Imaging. J. Am. Soc. Echocardiogr..

[13] Torri F., Lopriore P., Montano V., Siciliano G., Mancuso M., Ricci G. (2023). Pathophysiology and Management of Fatigue in Neuromuscular Diseases. Int. J. Mol. Sci..

[14] Philippou A., Xanthis D., Chryssanthopοulos C., Maridaki M., Koutsilieris M. (2020). Heart Failure-Induced Skeletal Muscle Wasting. Curr. Heart Fail. Rep..

[15] Zizola C., Schulze P.C. (2013). Metabolic and structural impairment of skeletal muscle in heart failure. Heart Fail. Rev..

[16] Gallagher H., Hendrickse P.W., Pereira M.G., Bowen T.S. (2023). Skeletal muscle atrophy, regeneration, and dysfunction in heart failure: Impact of exercise training. J. Sport Health Sci..

[17] Conte E., Imbrici P., Dinoi G., Boccanegra B., Lanza M., Mele E., Riemma M.A., Urbanek K., Cappetta D., De Luca A. (2025). SGLT2 inhibitor dapagliflozin mitigates skeletal muscle pathology by modulating key proteins involved in glucose and ion homeostasis in an animal model of heart failure. Eur. J. Pharmacol..

[18] Wood N., Straw S., Cheng C.W., Hirata Y., Pereira M.G., Gallagher H., Egginton S., Ogawa W., Wheatcroft S.B., Witte K.K. (2024). Sodium–glucose cotransporter 2 inhibitors influence skeletal muscle pathology in patients with heart failure and reduced ejection fraction. Eur. J. Heart Fail..

[19] Scullin D., Barney J. (2025). Evaluation of Neuromuscular Disease in Adults Presenting with Dyspnea. Semin. Respir. Crit. Care Med..

[20] Carluccio E., Biagioli P., Reboldi G., Mengoni A., Lauciello R., Zuchi C., D’aDdario S., Bardelli G., Ambrosio G. (2023). Left ventricular remodeling response to SGLT2 inhibitors in heart failure: An updated meta-analysis of randomized controlled studies. Cardiovasc. Diabetol..

[21] Perea-Armijo J., López-Aguilera J., Sánchez-Prats R., Castillo-Domínguez J.C., González-Manzanares R., Ruiz-Ortiz M., Mesa-Rubio D., Anguita-Sánchez M., Working Group of the Heart Failure Unit of Reina Sofia Hospital (2023). Improvement of Left Ventricular Ejection Fraction in Patients with Heart Failure with Reduced Ejection Fraction: Predictors and Clinical Impact. Med. Clin..

[22] Usman M.S., Siddiqi T.J., Anker S.D., Bakris G.L., Bhatt D.L., Filippatos G., Fonarow G.C., Greene S.J., Januzzi J.L., Khan M.S. (2023). Effect of SGLT2 Inhibitors on Cardiovascular Outcomes Across Various Patient Populations. J. Am. Coll. Cardiol..

[23] Bauersachs J., Soltani S. (2023). Sodium-glucose co-transporter 2 inhibitors and mineralocorticoid receptor antagonists synergism in heart failure: It takes two to tango. Eur. Heart J..

[24] Battistoni I., Pongetti G., Falchetti E., Giannini I., Olivieri R., Gioacchini F., Bonelli P., Contadini D., Scappini L., Flori M. (2024). Safety and Efficacy of Dapagliflozin in Patients with Heart Failure with Reduced Ejection Fraction: Multicentre Retrospective Study on Echocardiographic Parameters and Biomarkers of Heart Congestion. J. Clin. Med..

